# Reliable Aerial Mobile Communications with RSRP & RSRQ Prediction Models for the Internet of Drones: A Machine Learning Approach

**DOI:** 10.3390/s22155522

**Published:** 2022-07-24

**Authors:** Mehran Behjati, Muhammad Aidiel Zulkifley, Haider A. H. Alobaidy, Rosdiadee Nordin, Nor Fadzilah Abdullah

**Affiliations:** Department of Electrical, Electronics and Systems Engineering, Faculty of Engineering and Built Environment, Universiti Kebangsaan Malaysia, Bangi 43600, Malaysia; mehran.behjati@ukm.edu.my (M.B.); aidiel.zulkifley@gmail.com (M.A.Z.); p92976@siswa.ukm.edu.my (H.A.H.A.); fadzilah.abdullah@ukm.edu.my (N.F.A.)

**Keywords:** UAV, drone, cellular connected, cellular communications, machine learning, channel modeling, RSRP, RSRQ

## Abstract

The unmanned aerial vehicle (UAV) industry is moving toward beyond visual line of sight (BVLOS) operations to unlock future internet of drones applications, including unmanned environmental monitoring and long-range delivery services. A reliable and ubiquitous mobile communication link plays a vital role in ensuring flight safety. Cellular networks are considered one of the main enablers of BVLOS operations. However, the existing cellular networks are designed and optimized for terrestrial use cases. To investigate the reliability of provided aerial coverage by the terrestrial cellular base stations (BSs), this article proposes six machine learning-based models to predict reference signal received power (RSRP) and reference signal received quality (RSRQ) based on the multiple linear regression, polynomial, and logarithmic methods. In this regard, first, a UAV-to-BS measurement campaign was conducted in a 4G LTE network within a suburban environment. Then, the aerial coverage was statistically analyzed and the prediction methods were developed as a function of distance and elevation angle. The results reveal the capability of terrestrial BSs in providing aerial coverage under some circumstances, which mainly depends on the distance between the UAV and BS and flight height. The performance evaluation shows that the proposed RSRP and RSRQ models achieved RMSE of 4.37 dBm and 2.71 dB for testing samples, respectively.

## 1. Introduction

Unmanned aerial vehicles (UAVs), also known as drones, are one of the fastest emerging technologies. Recently, low-altitude UAVs have remarkably received tremendous attention for civil applications, such as surveillance, transportation, environmental monitoring, industrial monitoring, agriculture services, disaster rescue, and goods and medical delivery [1,2,3,4,5,6].

Currently, in most parts of the world, drone applications are limited to operating within pilots’ visual line of sight [7]. However, for applications such as package delivery, drones are expected to operate autonomously in a long distance, where there is no visual line of sight as no pilot is observing drones during their missions. Hence, the next step in drone technology is to allow the drone to fly beyond visual line of sight (BVLOS).

On the other hand, to ensure a safe flight, drones need stable and reliable wireless connectivity for payload and control and command (CC) communications [7]. Generally, four wireless technologies can be considered for UAV communications: direct link, satellite, ad hoc network, and cellular network [8]. Each has its advantages and disadvantages.

Conventionally, most drones operate on the license-free industrial, scientific, and medical (ISM) radio band (2.4 GHz) as a direct link. Although establishing this link is low cost and simple, it suffers from low range, low data rate, and vulnerability to interference. Hence it is not suitable for BVLOS applications. Although satellite links can provide glob-al coverage, their equipment is costly, heavy, and energy-consuming. In addition, a satellite link suffers from high latency and large signal attenuation, where these features make satellite communication unsuitable for most drone use cases. An ad hoc network, on the other hand, is considered a robust and adaptable wireless technology. Nevertheless, it suffers from technical issues such as low spectrum efficiency, intermitted connectivity, and complex routing protocols [8].

Among the existing wireless technologies, the cellular network has recently been considered one of the main enablers for large-scale UAV communications [9]. In addition, the terrestrial cellular networks already exist; hence, there is no need to develop a new dedicated infrastructure for UAV wireless communications.

The main question is whether a cellular network can provide a reliable communication link to ensure that UAVs can fly BVLOS safely. In general, the existing 4G and 5G cellular networks provide ubiquitous coverage with low latency and a high-speed data rate for terrestrial users [10], whereas, at the altitudes below base stations’ (BSs) heights, the existing cellular networks can meet the CC and payload communication requirements to provide safe mobile connectivity for UAVs [11].

However, existing cellular networks are designed for terrestrial users and providing connectivity to the flying drone is challenging. On the one hand, with increasing height above the ground, the radio environment changes, and some problems arise, such as handover (HO) and mobility management [12] and severe interference between aerial users (UAVs) and terrestrial users [13]. On the other hand, the transmitting antennas are tilted down; therefore, there is a severe reduction in the antenna gain at higher altitudes, leading to lower link reliability and data rate [14].

**Figure 1:**
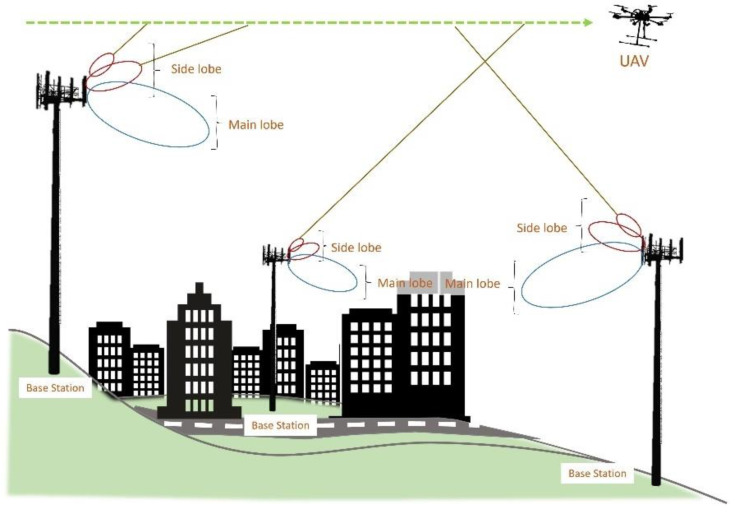
Illustration of cellular-connected UAV.

Figure 1 illustrates a scenario when a low-altitude UAV flies over the terrestrial BSs. By enhancing the flight height, the probability of line-of-sight (LoS) communications in-creases. Although attenuation factors, such as path loss and large-scale fading, are lower in LoS communications, in altitudes above the BS antennas’ height, the drone communications suffer from both uplink and downlink interferences [15], as in higher altitudes, the drone is in LoS of adjacent/interfering cells as well.

On the other hand, cellular networks are optimized for terrestrial UEs, e.g., BS antennas are tilted downward to prevent inter-cell interference and provide service for the UEs via antennas’ main lobes [16]. Meanwhile, by increasing the drone flight height, the probability of being served by the antennas’ sidelobes will increase, causing severe challenges, such as degradation of the received signal strength and quality [14], and increasing the frequency of HO and outage probability [9].

To further study the performance and quality of the communication link between the terrestrial BSs and UAVs, this article aims to propose machine learning (ML)-based models for predicting reference signal received power (RSRP) and reference signal received quality (RSRQ) in UAV-to-BS communications. It should be noted that, in this study, it was decided to use RSRP and RSRQ instead of path loss to manifest the final output in a more meaningful way. Both RSRP and RSRQ are key parameters directly representing the state of network signal level and quality at the UE location in 4G/LTE and 5G NR networks [17]. Additionally, modeling path loss requires knowledge about eNB parameters, including antenna gain, azimuth, feeder loss, power, etc. The latter are mostly difficult to obtain because of several technical and security concerns. Accordingly, a measurements campaign was conducted in a suburban environment to measure RSRP, RSRQ, and other important network parameters under different distances and flight heights. Then, the collected data were preprocessed and analyzed to investigate the cellular-connected UAV system’s performance under different considered scenarios. Finally, ML techniques have been used to develop the prediction models for RSRP and RSRQ.

The key contributions of this study are summarized as follows:

- A comprehensive measurement campaign has been conducted in a 4G LTE network within a suburban environment, consisting of about 28,000 physical layer samples. The performed aerial drive test focused on mobile link reliability between UAV and terrestrial BS. Hence, parameters such as RSRP, RSRQ, latency, and handover were measured.
- An open-source dataset has been provided, which is publicly accessible at [18]. The dataset contains about six hours of aerial drive tests under different measurement scenarios such as routes, BS, BSs’ heights, and UAV’s height in a harsh tropical suburban environment.
- Performance of the cellular-connected UAV system in the commercial LTE network has been investigated in a 3D form, under different distances and flight heights, in terms of RSRP and RSRQ. The statistical analysis reveals the performance of terrestrial BS in providing aerial coverage under different distances and flight heights. The output of this stage can be extended for other UAV mobile connectivity research, such as UAV path planning optimization.
- Six ML-based models have been considered and evaluated for an accurate RSRP and RSRQ prediction for LTE networks in suburban environments. Multiple linear regression, polynomial, and logarithmic methods were utilized to estimate the level of RSRP and RSRQ based on the 2D distance between the drone and serving BS, elevation angle, and flight height.

The rest of the paper is organized as follows:

A review of recent related works is presented in Section 2. Section 3 describes the materials and methods used in this research, including measurement methodology, methods to analyze and assess the collected data, and the utilized method and steps toward developing RSRP and RSRQ prediction models. Measurement and simulation results are presented and discussed in Section 4. Finally, the paper ends with a conclusion in Section 5.

## 2. Related Works

Recently, plenty of studies have been conducted to investigate the performance of existing 4G/LTE networks for UAV operations. One of the most precise and significant studies was performed by the 3rd Generation Partnership Project (3GPP), as reported in [17] and summarized in [19]. 3GPP formed a team in the “Radio Access Network studying Enhanced LTE Support for Aerial Vehicles” and performed numerous flight tests and simulations to evaluate the use of 4G LTE as a potential communication solution for UAV operation. Accordingly, the researchers verified the system performance level and identified the density of supportable heights, speeds, and UAVs. In addition, air-to-ground channel models were developed for different scenarios, and performance-enhancing solutions were studied for interference detection and mitigation, handover, and positioning.

In addition, some recent works in [13,15,20,21] investigated the performance of cellular-connected drones in simulation environments under different flight heights and drone speed scenarios. Accordingly, different measurement metrics were considered, including signal-to-interference plus noise ratio (SINR), RSRP, and HO. The results showed that the probability of LoS communication links increases by increasing the flight altitude, which, on the one hand, reduces the destructive effects such as penetration loss and shadowing and, on the other hand, enhances the inter-cell interference level, especially on the uplink. In addition, the papers demonstrated how drones are served by BS antennas’ sidelobes and revealed the issue of HO when drones move to the BS antenna sidelobe nulls. However, the conducted studies were limited to simulation results and therefore did not fully reflect real-world scenarios’ technical challenges and constraints.

Other research works, such as [12,13], performed field measurements to investigate the performance of cellular-connected drones in different scenarios. The performance of drone connectivity in an LTE network was investigated in terms of coverage, data-rate, interference, and latency. Results showed that existing LTE networks could provide communication links for low-altitude drones. However, the results were limited to specific scenarios, such as remote or rural environments. The authors in [22] performed a set of aerial drive tests in a suburban area to investigate the performance of LTE for UAV communications. It was found that the existing LTE network can provide aerial coverage under some circumstances, which mainly depends on the position of the UAV to the BS.

The authors in [23] conducted a measurement campaign in the 4G network and the result evaluation shows the weakness of the existing cellular networks in providing areal coverage, compared to terrestrial applications. In addition, the authors proposed a long-range multi-link communication system for UAVs, in which the developed communication module leverages multiple LTE modems and networks together with a multipath transmission control protocol for multi-link aggregation. The results show that the pro-posed system can considerably increase the communication link availability in maritime scenarios and provide smooth handover between different networks.

Besides performance evaluation, recently, the topic of UAV-to-BSs channel modeling has attracted the attention of researchers and, thereby, plenty of works have been con-ducted on this domain. For instance, ref. [24] reviewed the recently developed state-of-the-art air-to-ground channel models for different wireless communication technologies, including cellular-connected UAVs. Meanwhile, ref. [25] developed an empirical channel model for the UAV-to-BS scenario based on path loss and shadowing effects in the LTE network. The results showed that the path loss exponents decrease by increasing the flight height. The research findings also revealed the need for a height-based channel model for describing the propagation channel between UAVs and BSs.

It should be noted that based on the antenna radiation pattern, the gain of the antenna degrades by moving from the center of the main lobe to its boundaries. The amount of degradation depends on the design characteristics of the antenna. Hence, conventional 2D channel models, which are mainly based on the distance between transmitter and receiver, are not anymore suitable for aerial mobile communications, especially for UAV communications where the drones fly at different heights and, in the case of cellular-based communications, mainly receive signals from the boundaries of the main lobe or side lobes. Therefore, the development of 3D channel models for describing the propagation channel between UAVs and BSs is highly vital and demanding.

Authors in [26] provided an insight into the propagation characteristics of the UAV-to-BS channel and proposed a statistical path loss model for suburban environments. The proposed path loss model is a function of the depression angle and the terrestrial coverage beneath the UAV. In contrast, ref. [27] followed a different approach by proposing an ML-based channel model for the UAV-to-BS scenario. The model was developed based on the received signal strength (RSS) and an unsupervised clustering algorithm, in which the drone can identify the status of the current channel without relying on statistical channel models. However, the proposed method was simply a function of distance only and did not consider the effect of the UAV’s height or elevation angle.

In contrast, ref. [28,29,30] trained different ML models for predicting either the RSS or RSRP of UAV-to-BS using a total of nine features. These features included the latitude and longitude of the UAV and the nearest BS, the ground elevation and altitude of the UAV, the ground elevation and building height of the BS, and the antenna mast height of the BS. In [29], the authors utilized multiple ensemble learning methods to predict the RSS at several heights (up to 350 m) in an urban environment. In addition, they presented a new ensemble method based on five base learners: support vector machine (SVM), Gaussian processes (GP), artificial neural network (ANN), least-squares boosting (LSBoost), and bagging. The Salp swarm algorithm (SSA) was used to integrate base learners into the new method. Results showed that the proposed ensemble method was the best, with root mean squared error (RMSE) of 6.26 dB, mean absolute error (MAE) of 3.54 dB, and mean absolute percentage error (MAPE) of 3.92%.

Similarly, ref. [30] evaluated k-nearest neighbors (kNN), support vector regression (SVR), random forest (RF), AdaBoost, and gradient tree boosting (GTB) models for predicting RSRP at different UAV heights in an LTE network operating at 1800 MHz in urban and suburban environments. They introduced a new ensemble method that combines the latter base ML learners in an ensemble learning method termed voting regression (VR). The outcomes showed that the VR model outperformed the original base learners, achieving MAE of 3.227 dB, RMSE of 6.674 dB, and MAPE of 3.357%. Meanwhile, in [28], the authors presented a UAV-to-BS RSRP prediction model based on ANN for LTE networks operating at 1800 MHz frequency band in an urban environment, concerning UAV heights of up to 350 m. Combining the Levenberg–Marquardt (LM) backpropagation algorithm with self-adaptive differential evolution (DE) techniques led to the development of two novel hybrid training methods, namely the jDE and the composite DE (CoDE) algorithms. Both methods showed favorable outcomes, with CoDE-LM achieving the best.

In [31], a deep ANN was used to predict RSRP and RSRQ for the UAV-to-BS communication scenario in a rural LTE network based on the spatial positioning of a UAV flying up to 180 m in altitude. This approach takes the UAV’s position as input and maps them to seven training features, including latitude, longitude, altitude, azimuth, elevation, and radius. Results showed that the model performed decently with a cost function of 0.3 dB for training data and 0.4 dB for validation data when predicting RSRP. However, there are major limitations that are mainly due to considering one BS in a rural environment, lacking other communication scenarios, and using limited training/testing datasets.

Finally, we summarize the reviewed studies in Table 1 with a detailed description of the research focus, modeling approaches, key findings, and limitations of each study. In conclusion, it can be noted that most of the conducted studies tend to be limited to specific study areas and use positional data, latitude and longitude, as input features to the models. The latter, in turn, makes these models site-specific, i.e., they perform poorly when used in areas different than the study area, thereby not being reusable for other deployment environments/scenarios. Hence, we believe that still more research needs to be conducted to identify the radio propagation characteristics of a UAV-to-BS channel under different scenarios and use cases.

## 3. Methodology

The following subsections describe in detail each of the methods used in this study.

### 3.1. Field Trial Measurement

To develop realistic prediction models that reflect the real mobile radio propagation characteristics, we first conducted a set of aerial drive tests and then used the measurements to evaluate the cellular link performance in a UAV-to-BS communication scenario. The following describes the measurement setup/methods, study area, and validation strategies for the results.

**Figure 2:**
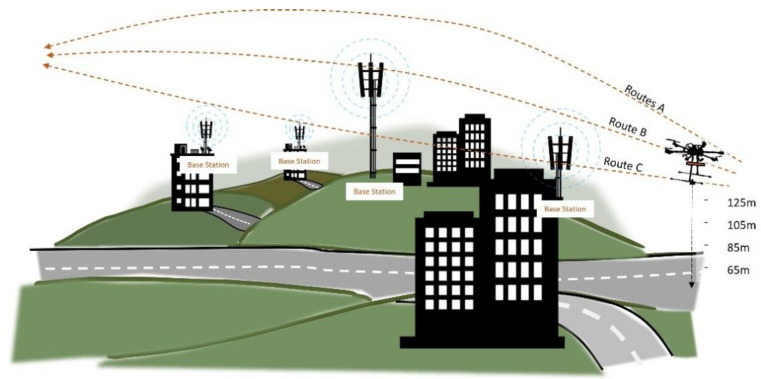
Illustration of the drive test method in three different routes, A-C, and four different heights, 65–125 m.

Figure 2 depicts the general concept of the utilized measurement method. The developed multirotor drone in [24] has been used to perform the drive-test. For measuring cellular key performance indicators (KPIs), a smartphone with an installed drive-test application is required. Plenty of drive-test applications are available on Google Play Store for Android devices, such as GNet Track Pro [32], RF Signal Tracker [33], and Network Cell Info [34]. In this study, the GNet Track Pro has been used due to its accuracy and capability to measure and monitor the essential KPIs of the cellular network compared to other existing applications [24].

**Figure 3:**
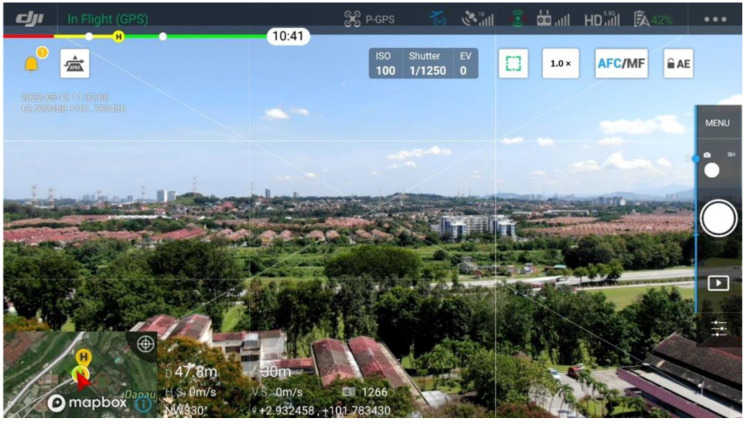
Overview of the suburban study environment for the 4G dataset measurement.

The measurement was conducted at the National University of Malaysia (UKM). The campus environment can be considered a tropical suburban metropolitan area with a geographic terrain of undulating hills and dense vegetation. Figure 3 shows an aerial image of the considered area.

In the considered study area, cellular coverage is provided by different telecommunication service providers and, in this study, only one of the cellular operators with better coverage was selected for the measurement. The measurement area is covered by four LTE BSs with a carrier frequency of 2.6 GHz and a system bandwidth of 20 MHz. Table 2 presents the information on BSs.

The smartphone was mounted on the drone and served by the LTE network during the measurement. At the same time, GNet measured the LTE-related parameters such as drone position, RSRP, RSRQ, and network latency. The measured KPIs were then stored on the phone and retrieved after landing. It should be noted that during the tests, the drone speed was maintained at an average speed of 20 km/h.

Drive tests were conducted on three different paths and four elevations (65, 85, 105, and 125 m). Meanwhile, to enhance measurement accuracy, each flight set was repeated twice. Moreover, the starting point’s elevation was considered the reference point, about 40 m above sea level. For example, flying at an altitude of 65 m means flying at an altitude of 65 m above the starting point or equivalent to 105 m above sea level.

### 3.2. ML-Based RSRP and RSRQ Prediction Models

To develop a realistic prediction model to represent the performance of an LTE communication link based on the capability of the GNet track, RSPR and PRSQ parameters were selected as the parameters to be measured and modeled. RSRP is a key measurement parameter indicating the average received signal power of a single resource element in an LTE resource block (RB) and can be calculated as [17]:

(1) $$\mathrm{RSRP}\left[W\right]=\frac{1}{N}\overset{N}{\underset{n=1}{\sum }}P_{n\;},$$

where N is the number of received reference signals and $P_{n\;}$ is the received power of *n*-th reference signal. However, RSRP alone does not fully reflect the quality of the received signal because it also picks up the energy of interfering signals in the corresponding frequency range.

RSRQ is considered another key measurement parameter that indicates the received signal quality level in the LTE network and the effect of interference from adjacent BSs. The RSRQ can be calculated as [17]:

(2) $$\mathrm{RSRQ}=N\times \frac{\mathrm{RSRP}\left[W\right]}{\mathrm{RSSI}\left[W\right]}\;,$$

where the reference signal strength indicator (RSSI) is the power measured over the entire bandwidth of occupied RBs, including intracell power, interference, and noise. RSRQ is dimensionless and usually written in dB. Table 3 demonstrates the status of a signal based on its measured RSRP and RSRQ level [35].

**Figure 4:**
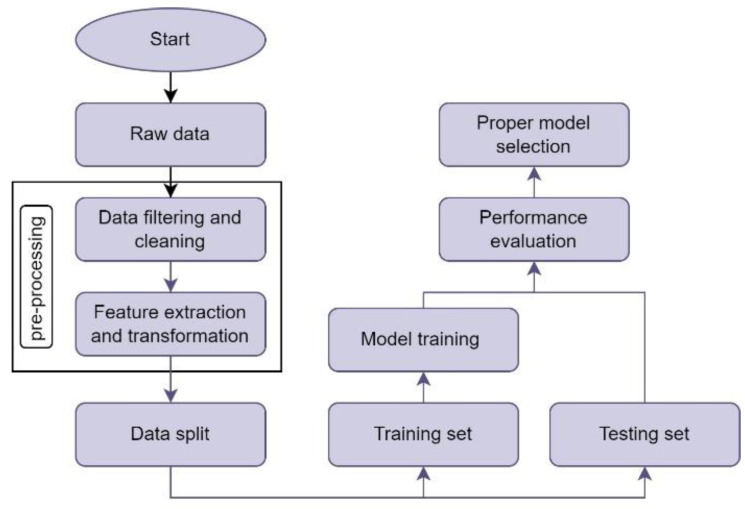
Flowchart of the ML-based RSRP and RSRQ modeling.

The *NumPy* [36] and *Pandas* [37] libraries were used to clean, preprocess, and analyze the collected data statistically. Figure 4 depicts the flowchart of the ML-based RSRP and RSRQ modeling. The raw data consists of a 28,389 × 42 matrix, where each row represents one measured sample, including the drone’s geographical location, sampling time, and information about the cellular network and received signals. First, the raw data were filtered to extract the measured samples and required features from the desired BSs. In addition, the measurements were cleaned by removing outliers and irregular measurement points. Then, as independent variables, two new columns were added to the dataset, which are 2D distance and elevation angle. The 2D distance, $d_{2D}$, was measured based on the GPS coordinates of the serving BS and the drone at each sampling point, as [38]:

(3) $$d_{2D}=2\times ℛ\times \arctan2\left(\sqrt{𝒶},\;\sqrt{1-𝒶}\right),$$

(4) $$a=sin^{2}\left(\frac{lat_{BS}-lat_{UAV}}{2}\right)+\cos\left(lat_{\mathrm{BS}}\right)\times \cos\left(lat_{\mathrm{UAV}}\right)\times \sin^{2}\left(\frac{lon_{\mathrm{BS}}-lon_{\mathrm{UAV}}}{2}\right)\;,$$

where ℛ is the Earth’s mean radius equal to 6371 km, $lat_{\mathrm{BS}}$ and $lon_{\mathrm{BS}}$ are the latitude and longitude of the serving BS in the decimal degrees format, respectively. $lat_{\mathrm{UAV}}$ and $lon_{\mathrm{UAV}}$ are the latitude and longitude of the drone in considered sampling point, respectively.

Elevation angle, β, was calculated based on the height of BS’s antennas, $h_{\mathrm{BS}}$, flight altitude, $h_{\mathrm{UAV}}$, and $d_{2D}$, as:

(5) $$\beta =\arctan\left(\frac{h_{\mathrm{UAV}}-h_{\mathrm{BS}}}{d_{2D}}\right)+\alpha ,$$

where α is the tilt angle of the BS’s antenna as listed in Table 1. Note that the UAV flight height was always above the BSs’ height in the measurement scenarios. Hence, the divisor in the above equation is always a positive number. In this step, to accelerate modeling speed, improve accuracy, prevent bias, and avoid other scale difference issues in the model fitting phase, the determined features were normalized.

After preprocessing the data, the dataset contains the following information for 8457 samples: cell ID, 2D distance, elevation angle, RSRP, and RSRQ. Out of the latter, RSRP and RSRQ are considered the dependent variables, i.e., the goal of prediction.

At this stage, ML was used to predict the RSRP and RSRQ values at different heights and distances. Supervised ML algorithms can look at independent variables in a dataset and predict a dependent variable based on the characteristics of independent variables, i.e., predict trends by using previously labeled data. In this study, the *Scikit* library [39] was used for ML model training and evaluation. Since there are multiple independent variables and based on the distribution of the collected data, different methods have been used to develop prediction models for RSRP and RSRQ. These methods include LARS lasso, SVR, polynomial, and logarithmic methods.

The LARS lasso method is implemented based on the LARS algorithm, which provides the full path of the coefficients along with the regularization parameter, and unlike the coordinated descent-based algorithm, yields an exact solution. SVR extends the support vector machine (SVM) method for solving regression problems. There are different implementations/kernels of SVR, such as epsilon, nu, and linear SVR. The fit time complexity of SVR kernels is high, e.g., in epsilon implementation, it is more than quadratic with the number of samples. Hence, based on the size of our dataset, the linear kernel has been selected for model training. The trained models by the LARS lasso and SVR methods can be represented as:

(6) $$\mathrm{RSRP}\;or\;\mathrm{RSRQ}=\theta_{0}+\theta_{1}\times d_{2D}+\theta_{2}\times \beta ,$$

where $\theta_{0}$ is the intercept and $\theta_{1}$ and $\theta_{2}$ are coefficients that predict the impact of change on the $d_{2D}$ and β, respectively.

One common strategy within ML is to use linear models trained on nonlinear functions of the data. This strategy maintains the fast performance of linear methods while letting them fit a broader range of data. The generated model with this approach depends on the degree of polynomials. For example, for second-order polynomials, the model can be presented as:

(7) $$\mathrm{RSRP}\;or\;\mathrm{RSRQ}=w_{0}+w_{1}\times d_{2D}+w_{2}\times \beta +w_{3}\times d_{2D}^{2}+w_{4}\times d_{2D}\times \beta +w_{5}\times \beta^{2},$$

where $w_{0},\;\ldots ,\;w_{5}$ are the polynomial coefficients that are calculated and optimized by the utilized algorithm.

However, for the cases where the data shows a curvy trend, the linear regression cannot produce very accurate results when compared to nonlinear regression. Therefore, as the third method, the logarithmic model has been used to model the nonlinear relationship between the independent variables, distance, and elevation angle, and the dependent variables, RSRP and RSRQ. The reason for choosing the logarithmic model is that, based on the data distribution, a logistic function can provide a good approximate model, since it has the property of being fit with the changes in the dataset. In addition, it is consistent with the well-known conventional logarithmic models such as Okumura-Hata, COST231-Hata, and ITU-R P.1546. Therefore, the nonlinear models have been fitted based on the following equations:

(8) $$\mathrm{RSRP}\;or\;\mathrm{RSRQ}=\varphi_{1}+\varphi_{2}\times \log_{10}\left(d_{2D}\right)+\varphi_{3}\times \log_{10}\left(\beta \right),$$

where $\varphi_{1},\;\varphi_{2}$, and $\varphi_{3}$ are the coefficients that can be calculated and optimized by the *curve_fit* function from the *optimize* module of the *SciPy* library.

The dataset was randomly split into train and test sets with a portion of 80% and 20%, respectively. The train set was used to train the model, and the test set was used to evaluate the accuracy of the prediction model.

The “*metrics*” module of *Scikit-learn* has been used to measure the performance of regression models. In this study, metrics such as RMSE, MAPE, and median absolute error (MedAE) were used to evaluate the models’ performances.

RMSE is the standard deviation of the prediction errors and residuals. Residuals are a measure of how far from the regression line data points are and can be estimated over *n* samples as:

(9) $$\mathrm{RMSE}=\sqrt{\frac{1}{n}\sum_{j=1}^{n}{\left(y_{j}-{\hat{y}}_{j}\right)}^{2}},$$

where ${\hat{y}}_{j}$ is the predicted value of the *j*th sample and $y_{j}$ is the corresponding true value.

MAPE, also known as mean absolute percentage deviation, is a measure of prediction accuracy of a statistical forecasting model and expresses the accuracy as a percentage, which can be calculated as:

(10) $$\mathrm{MAPE}=\frac{1}{n}\sum_{j=1}^{n}\frac{\left|y_{j}-{\hat{y}}_{j}\right|}{\max\left(\epsilon ,\left|y_{j}\right|\right)},$$

where ϵ is an arbitrarily small positive number to avoid undefined results when $y_{j}$ is zero.

MedAE is a robust measure of the variability of a univariate sample of quantitative data, and it is particularly interesting because it is robust to outliers. The loss is calculated by taking the median of all absolute differences between $y_{j}$ and ${\hat{y}}_{j}$, as:

(11) $$\mathrm{MedAE}=median\left(\left|y_{1}-{\hat{y}}_{1}\left|,\;\ldots ,\;\right|y_{n}-{\hat{y}}_{n}\right|\right).$$

## 4. Results and Discussion

This section summarizes the collected data from the 4G LTE drive test. Then, it investigates the aerial cellular coverage and analyzes the data in different scenarios. Finally, the results of the developed RSRP and RSRQ prediction models are discussed.

### 4.1. Measurement Results and Analysis

**Figure 5:**
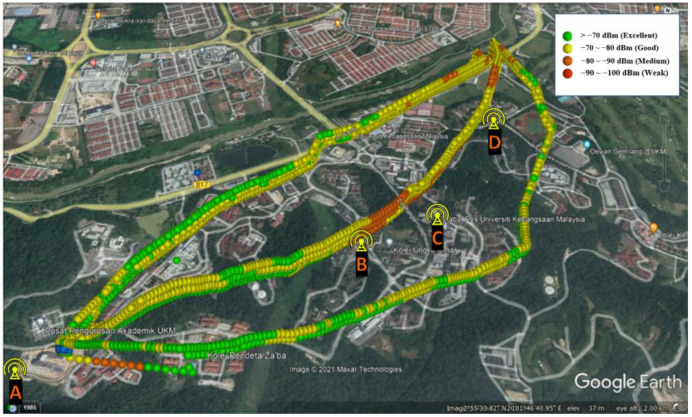
Measured RSRP values at different routes and elevations.

**Figure 6:**
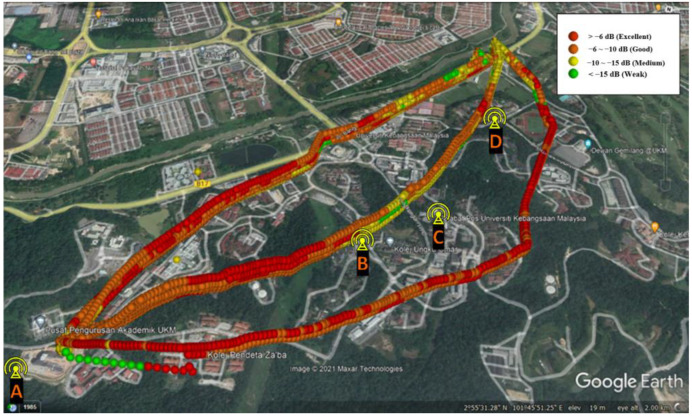
Measured RSRQ values at different routes and elevations.

Figure 5 and Figure 6 show the overall drive test results for different paths and heights in terms of RSRP and RSRQ, respectively. The flight distance for routes A, B, and C are about 2.3, 2.4, and 2.6 km, respectively, and the flight heights are 65, 85, 105, and 125 m above the takeoff point. The colors indicate the RSRP and RSRQ conditions, which are changing in the “excellent” to “weak” range, based on Table 2. Based on the results, the link performance depends on the 3D position of the drone relative to BSs, where, generally, the shorter the distance and the lower the altitude, the better the performance.

During the drive tests, the mounted phone on the drone was served by 13 BSs. It was observed that the number of serving cells increased by increasing the flight height. The main reason behind this phenomenon is that the drone is mainly served by the sidelobes of BS’s antennas when flight height increases. Hence, in higher altitudes, where the drone is in visual line of sight of the adjacent BSs, the probability of serving by the sidelobes of a larger number of BSs increases. However, the serving time from most of the adjacent BSs is not long, and after a short distance, a handover occurs, and another BS serves the drone. In this regard, we only considered the data belonging to the dominant BSs with a sufficient number of measured samples. After preprocessing and data cleansing, 8457 samples were considered for data analysis.

**Figure 7:**
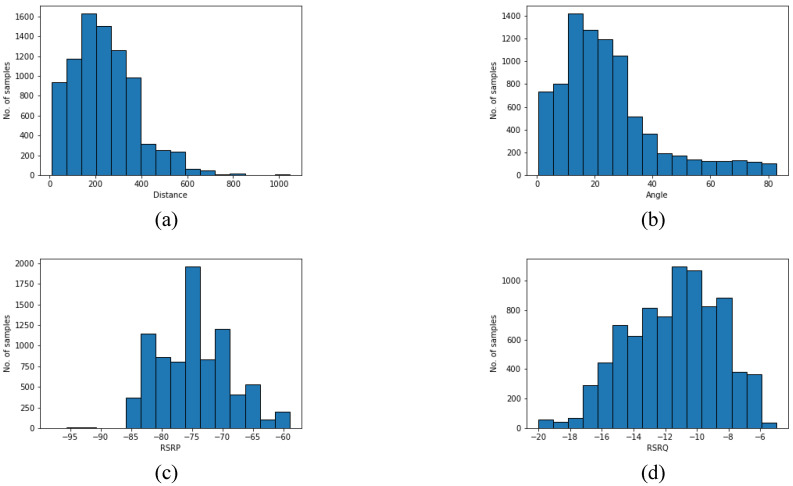
Histograms of dataset parameters, including (**a**) distance, (**b**) angle, (**c**) RSRP, and (**d**) RSRQ.

Table 4 describes a statistics summary of the considered data. Meanwhile, Figure 7 shows the histograms of distance, angle, RSRP, and RSRQ. Distance is the 2D distance between serving BS and the drone. The elevation angle is the angle between the drone’s position and the center of the antenna’s main lobe. Finally, height is the drone’s altitude above the serving BS’s antenna, and X% is the percentage of the data distribution.

**Figure 8:**
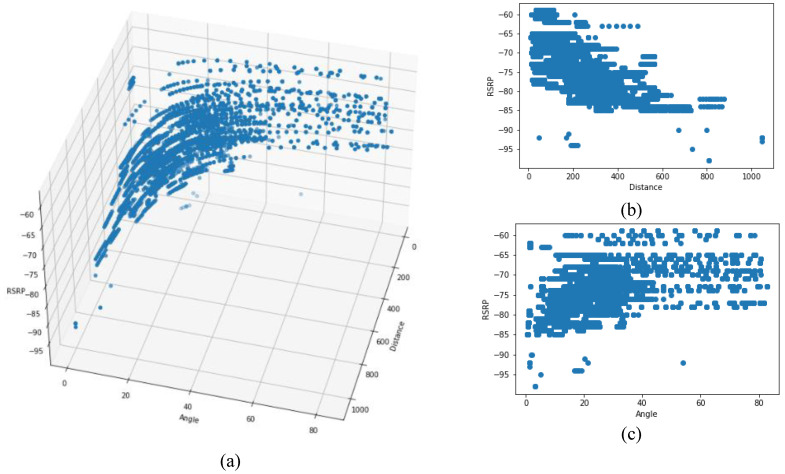
(**a**) Data distribution of RSRP versus angle and distance. (**b**) RSRP versus distance. (**c**) RSRP versus angle.

**Figure 9:**
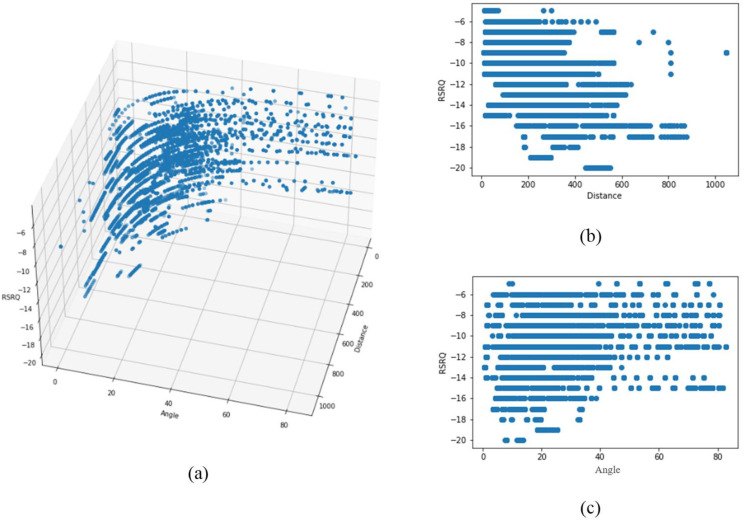
(**a**) Data distribution of RSRQ versus angle and distance, (**b**) RSRQ versus distance, and (**c**) RSRQ versus angle.

Figure 8a and Figure 9a show the 3D distribution of the measured RSRP and SRRQ values versus angle and distance, respectively. As the data shows, the performance of the cellular communication link directly depends on the distance between the drone and the serving BS, as well as the elevation angle or the flight height. For better visualization, Figure 8b,c and Figure 9b,c show the 2D plots of RSRP and RSRQ versus distance and angle, respectively. Results show that by increasing the distance and angle, both RSRP and RSRQ degrade, and degradation can be modeled with either linear or nonlinear models. Note, since the RSRP and RSRQ values depend highly on distance and angle, a 2D plot cannot reflect the impact of each factor on the link performance, so the data distribution seems to be scattered.

The results show that most data are distributed at less than 600 m and an elevation angle of fewer than 40 degrees. The reason for such a distribution is the handover and cellular network architecture in the considered measurement area, which is in line with the standard architecture of the cellular network in urban and suburban areas.

**Figure 10:**
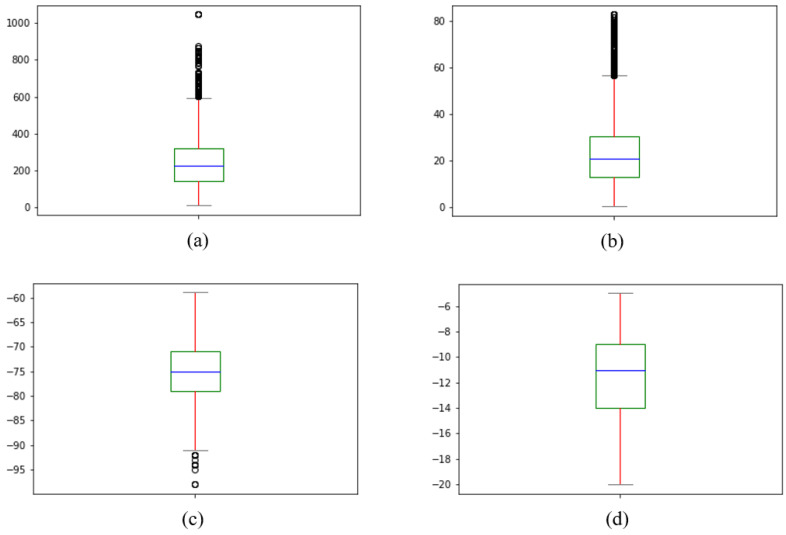
Box plots of (**a**) distance, (**b**) angle, (**c**) RSRP, and (**d**) RSRQ.

For a closer look, Figure 10 depicts the box plots of all collected data. From the results of Figure 10c,d, it can be seen that 75% of RSRP and RSRQ are in the range of −59 dBm and −79 dBm, and −5 to −14 dB, respectively. Based on the results of Figure 10a,b and Table 3, it can be revealed that within a distance of 320 m and an elevation angle of 30 degrees, most probably a terrestrial BS can serve a drone with good or medium signal strength and quality.

The hollow circles also show the outliers, which the boxplot function of the *matplotlib* library has identified. This data mainly belongs to the measurements at the heights of 105 and 125 m, where the adjacent BS is in the drone’s line of sight and receives service for a short period. However, since these samples are reliable and represent the signal condition over a long distance, we keep and consider these samples for developing the prediction models.

In conclusion, the data analysis revealed that the reliability and performance of a terrestrial cellular link in aerial communication is a function of distance and elevation angle. Enhancing the distance destructing factors such as path loss and shadowing attenuate the energy of receive signals, RSRP, at the drone side. In contrast, the degradation of RSRQ is mainly affected by the elevation angle enhancement. The main reasons behind this phenomenon are reduced antenna gain and enhanced signal interference at higher altitudes, resulting in a lower signal-to-interference plus noise ratio (SINR).

The reason for the desired signal level degradation is that the BSs antennas are down tilted and, based on the antenna radiation pattern, by increasing the flight height, the chance of serving the drone with the main lobe decreases, which consequently degrades the received signal energy at the drone side. The reason for interference enhancement is because, in higher altitudes, a drone can see a larger number of BSs. Therefore, the probability of receiving signals from adjacent/interfering BSs increases. Consequently, the drone receives higher interference energy at higher altitudes.

### 4.2. RSRP and RSRQ Prediction Models

Table 5 and Table 6 present the performance of the RSRP and RSRQ prediction models under LARS lasso, SVR, polynomial, and logarithmic methods, respectively. Table 5 also compares the results obtained in this study against a few related works. Results show that the performance of the proposed models outperformed other related works. Meanwhile, the performance of LARS lasso and SVR are almost the same, while SVR involves high computational complexity and requires more computation power. Hence, LARS lasso is selected as a linear regression method with acceptable accuracy. The results of the polynomial and logarithmic methods show that the polynomial degree of 2 could present slightly better results than the linear methods and the logarithmic method outperforms linear regression models. That latter comes since it uses nonlinear functions to train the data and generates a model that fits a much wider range of data.

On the other hand, the performance of a polynomial method depends on its degree, in which higher degrees enhance the flexibility of the model to fit a broader range of data. However, the overfitting issue should be considered, where the generated method may fit perfectly with the existing data but may not predict accurate output for new data. In this regard, Table 7 compares the evaluation results of the polynomial-based RSRP model under different degrees. Results thereby show that increasing the degree to 6 improves the performance slightly. Then, due to the overfitting, the performance starts to degrade. Hence, due to the simplicity of the equation, we believe that a degree of 2 can provide accurate enough prediction methods for RSRP and RSRQ.

**Figure 11:**
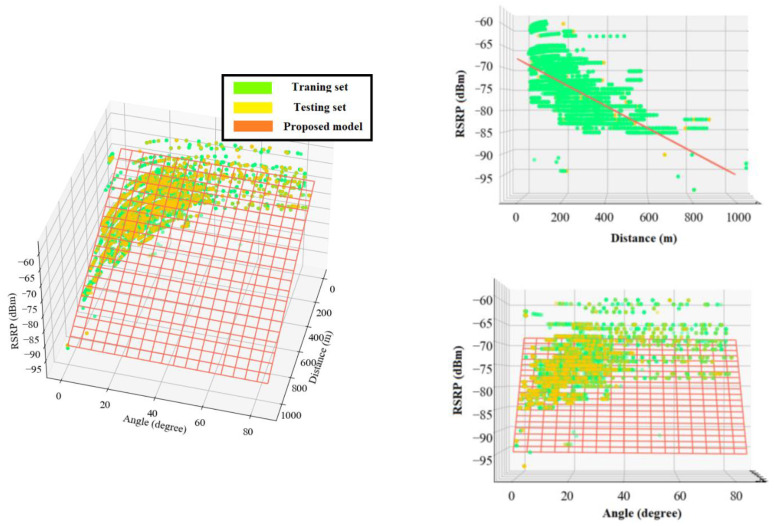
3D representation of the proposed linear regression model for RSRP.

**Figure 12:**
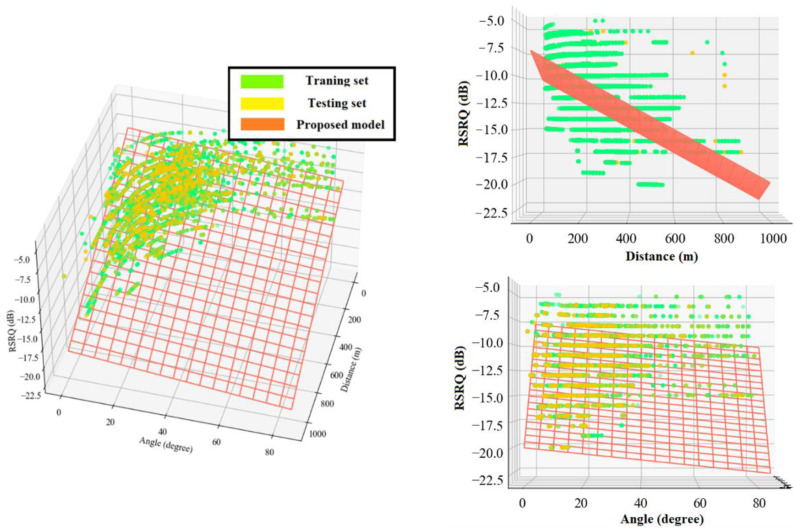
3D representation of the proposed linear regression model for RSRQ.

The following equations present the proposed multiple linear regression models for RSRP and RSRQ as a function of 2D distance in meter and elevation angle in degree. The angle coefficients showed that RSRQ is more angle-dependent than RSRP because of the higher interference level at higher altitudes. Figure 11 and Figure 12 depict a 3D representation of the linear regression-based RSRP and RSRQ prediction models, respectively.

(12) $${\mathrm{RSRP}}_{\mathrm{linear}}=-68.197-0.0264\times d_{2D}-0.0057\times \beta ,$$

(13) $${\mathrm{RSRQ}}_{\mathrm{linear}}=-7.7894-0.012\times d_{2D}-0.0279\times \beta \;.$$

**Figure 13:**
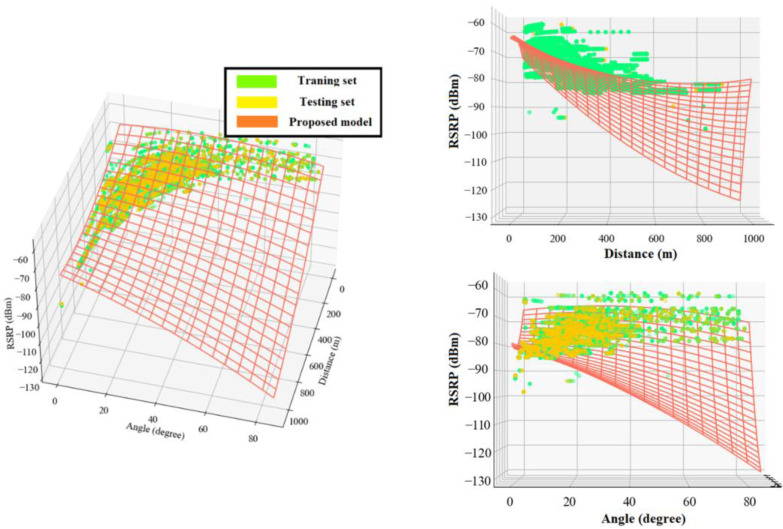
3D representation of the proposed polynomial model for RSRP.

**Figure 14:**
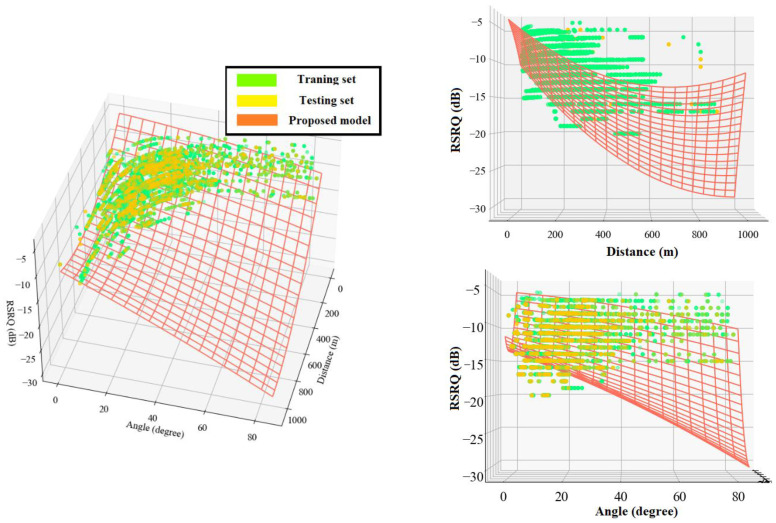
3D representation of the proposed polynomial model for RSRQ.

The following equations present the proposed polynomial-based RSRP and RSRQ prediction models, and Figure 13 and Figure 14 show a 3D representation of the proposed models for the RSRP and RSRQ, respectively.

(14) $${\mathrm{RSRP}}_{\mathrm{polynomial}}=-65.048-4.67\times 10^{-2}\times d_{2D}+0.11\times \beta +3.22\times 10^{-5}\times d_{2D}^{2}-4.35\times 10^{-4}\times d_{2D}\times \beta -2.04\times 10^{-3}\times \beta^{2},$$

(15) $${\mathrm{RSRQ}}_{\mathrm{polynomial}}=-4.679-2.66\times 10^{-2}\times d_{2D}-3.83\times 10^{-2}\times \beta +1.95\times 10^{-5}\times d_{2D}^{2}-1.47\times 10^{-4}\times d_{2D}\times \beta -3.13\times 10^{-4}\times \beta^{2}.$$

**Figure 15:**
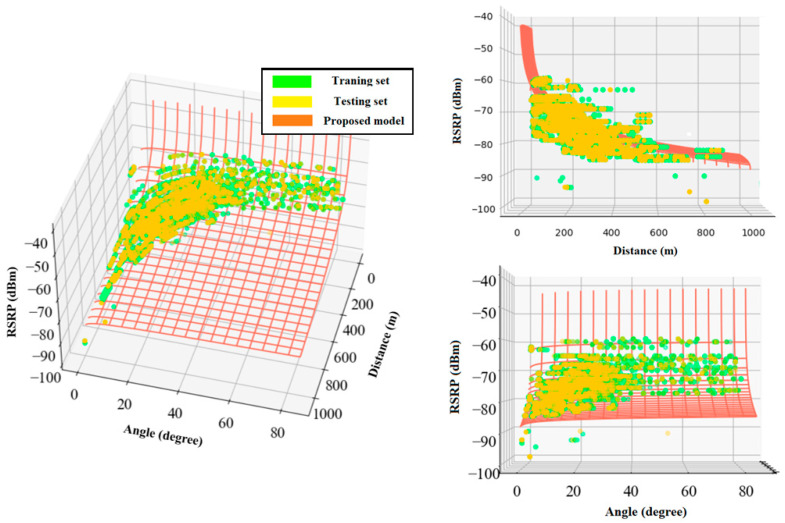
3D representation of the proposed logarithmic model for RSRP.

**Figure 16:**
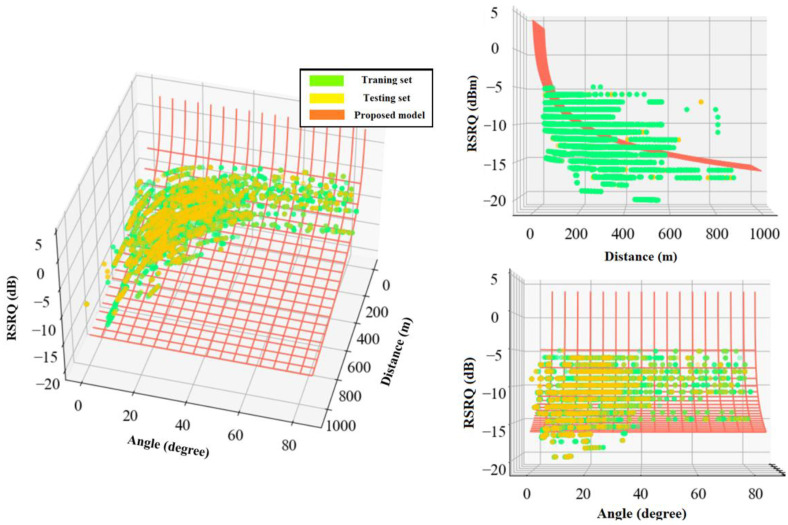
3D representation of the proposed logarithmic model for RSRQ.

Equations (16) and (17) present the proposed logarithmic-based models for RSRP and RSRQ, and Figure 15 and Figure 16 depict a 3D representation of the proposed models for RSRP and RSRQ, respectively.

(16) $${\mathrm{RSRP}}_{\mathrm{logarithmic}}=-30.75-8.11\times \log_{10}\left(d_{2D}\right)+0.07\times \log_{10}\left(\beta \right),$$

(17) $${\mathrm{RSRQ}}_{\mathrm{logarithmic}}=9.91-3.76\times \log_{10}\left(d_{2D}\right)-0.017\times \log_{10}\left(\beta \right).$$

Based on the presented results it can be concluded that all the proposed models can predict RSRP and RSRQ values with acceptable accuracy in suburban environments. The selection of a prediction model depends on its application; for example, for cases where computational resources are not the limiting factor and computational accuracy is critical, nonlinear models can be better choices, whereas linear models are simple to interpret and can provide a quick and accurate prediction.

## 5. Conclusions

In this work, we conduct an extensive measurement campaign in the 4G/LTE mobile network to investigate the performance of existing cellular networks in the low-altitude UAVs’ flight territory and develop ML-based prediction models for RSRP and RSRQ. According to the discussed results, it can be concluded that, although the existing LTE infrastructures are designed and optimized for terrestrial communications, the terrestrial BSs in suburban environments still can provide a communication link in a range of “good” to “moderate” conditions in terms of RSRP and RSRQ. It has been revealed that the reliability of the communication link is a function of the distance and the elevation angle, which depends on the cellular network design, flight height, surrounding environment, and terrain profile. The statistical analysis shows that the existing LTE networks are able to provide a reliable communication link for UAVs at a distance up to 400 m and elevation angle up to 30 degrees, while the RSRP is in the range of −59 dBm to −79 dBm and RSRQ is in the range of −9 dB to −14 dB. The results of RSRP and RSRQ modeling show that the logarithmic and polynomial models outperform the linear regression models because of the nonlinear curvature of data, in which the nonlinear models can accurately predict the considered metrics in a suburban environment up to a distance of 1 km and an elevation angle of 85 degrees. Finally, it was concluded that the proposed models outperform the existing models, and the selection of an appropriate prediction model, linear or nonlinear, depends on its application.

## Figures and Tables

**Table 1:** A summary of the reviewed UAV-to-BS channel modeling and characterization studies.

Ref.	Study Focus	Modeling Approach	Key Findings/Contributions	Limitations
[13,15,20,21]	Performance evaluation of cellular-connected drones	N/A	Demonstrated how drones are served by BS antenna sidelobes and revealed the issue of HO when drones move to the BS antenna sidelobe nulls.	Simulation-based and do not fully reflect real-world technical challenges and constraints.
[12,22,23]		N/A	The evaluation was based on field measurements. Showed that existing 4G LTE networks could provide communication links for low-altitude drones.	The evaluations were limited to specific communication scenarios, such as remote or rural environments.
[24]	Investigate LTE performance for UAV	N/A	Found that the existing LTE network can provide aerial coverage, constrained to the position of the UAV to the serving BS.	Limited to performance evaluation in suburban environments.
[25]	Survey existing and recently developed channel models	N/A	Reviewed recent state-of-the-art air-to-ground channel models for different technologies, including cellular-connected UAVs.	Limited to surveying existing models and recent developments for channel modeling.
[26]	UAV-to-BS channel modeling	Empirical path loss modeling for UAV-to-BS scenario.	The path loss exponents decrease by increasing the flight height, approximating free space propagation.	Limited to certain communication scenarios, utilizing conventional modeling techniques.
[27]		Statistical path loss modeling UAV-to-BS scenario.	The proposed path loss model is a function of the depression angle and the terrestrial coverage beneath the UAV.	Limited for suburban environments.
[28]	UAV-to-BS RSS modeling	ML-based modeling for RSS prediction.	-	The proposed method was simply a distance function, neglecting the effect of parameters such as the UAV’s height or elevation angle.
[29]	UAV-to-BS RSS modeling	ML-based (ensemble) modeling for RSS or RSRP prediction. Using nine input features.	They have utilized multiple ensemble learning methods to predict the RSS at several heights and presented a new ensemble method based on five base learners.	Limited to RSS prediction and uses latitude and longitude as input features to the models, making it a site-specific model.
[30]	UAV-to-BS RSRP modeling	ANN for RSRP prediction	Developed two hybrid training methods, the jDE and the CoDE algorithms. Both methods showed favorable outcomes, with CoDE-LM achieving the best.	Uses latitude and longitude as input features, making the proposed model site-specific.
[31]	UAV-to-BS RSRP and RSRQ modeling	Deep ANN for RSRP and RSRQ prediction.	Results showed that the model performed decently with a cost function of 0.3 dB for training data and 0.4dB for validation data when predicting RSRP.	Limited to one BS in a rural environment, lacking other communication scenarios and using limited training/testing datasets. Uses latitude and longitude as input features, making the proposed model site-specific.

**Table 2:** Physical information of the considered BSs.

	Type of BS	No. of Sectors	Tilt Angle (Degree)	Height (m) Above Ground Level	Height (m) Above Sea Level
**BS A**	Tower located on a hill	3 × 120°	6	28	99
**BS B**	Rooftop	3 × 120°	4	11	72
**BS C**	Rooftop	3 × 120°	4	23	56
**BS D**	Rooftop	3 × 120°	4	12	46

**Table 3:** Signal status based on RSRP and RSRQ value.

Signal Strength/Quality	RSRP	RSRQ
**Excellent**	−60~−70 dBm	>−6 dB
**Good**	−70~−80 dBm	−6~−10 dB
**Medium**	−80~−90 dBm	−10~−15 dB
**Weak**	−90~−100 dBm	<−15 dB

**Table 4:** Summary statistics of considered data.

	Distance (m)	Elevation Angle (Degree)	Height (m)	RSRP (dBm)	RSRQ (dB)
**mean**	242.195	24.534	79.547	−74.735	−11.396
**std**	141.782	17.066	28.396	5.832	3.081
**min**	10.534	0.560	6.000	−98.000	−20.000
**25%**	140.183	13.090	59.000	−79.000	−14.000
**50%**	224.761	21.040	89.000	−75.000	−11.000
**75%**	322.479	30.520	99.000	−71.000	−9.000
**max**	1045.209	82.700	119.000	−59.000	−5.000

**Table 5:** Performance of proposed prediction models for RSRP compared against related works.

Proposed Method/Reference	RMSE	MAPE (%)	MedAE	Notes
**LARS Lasso**	4.58	4.9	3.04	-
**SVR**	4.60	5.0	2.99	-
**Polynomial**	4.49	4.8	2.90	-
**Logarithmic**	4.37	4.6	2.81	-
[31]	6.26	3.92	-	Predicts RSS
[32]	6.674	3.357	-	-
[33]	9.63–12.32	-	-	-

**Table 6:** Performance of proposed prediction models for RSRQ.

Method	RMSE	MAPE (%)	MedAE
**LARS Lasso**	2.80	22	1.86
**SVR**	2.81	21	1.86
**Polynomial**	2.75	22	1.90
**Logarithmic**	2.71	21	1.87

**Table 7:** Performance of polynomial method under different degrees.

Polynomial Degree	RMSE	MAPE (%)	MedAE
**2**	4.49	4.8	2.90
**4**	4.33	4.6	2.82
**6**	4.31	4.6	2.80
**8**	4.39	4.7	2.84
**10**	4.48	4.8	2.97

## Data Availability

The data presented in this study are publicly available on: Mehran Behjati, 2022, UAVCellularDataset_suburban, GitHub, https://github.com/Mehranbjt/UAVCellularDataset_Suburban (accessed on 17 July 2022).
